# Detection of Alternative Splice and Gene Duplication by RNA Sequencing in Japanese Flounder, *Paralichthys olivaceus*

**DOI:** 10.1534/g3.114.012138

**Published:** 2014-11-05

**Authors:** Wenji Wang, Jing Wang, Feng You, Liman Ma, Xiao Yang, Jinning Gao, Yan He, Jie Qi, Haiyang Yu, Zhigang Wang, Xubo Wang, Zhihao Wu, Quanqi Zhang

**Affiliations:** * College of Marine Life Sciences, Ocean University of China, Key Laboratory of Marine Genetics and Breeding, Ministry of Education, Qingdao, 266003, China; † School of Life Science, Taizhou University, Taizhou, 318000, China; ‡ Key Laboratory of Experimental Marine Biology, Institute of Oceanology, Chinese Academy of Sciences, Qingdao, 266071, China

**Keywords:** *Paralichthys olivaceus*, transcriptome, alternative splicing, gene duplication, double haploids

## Abstract

Japanese flounder (*Paralichthys olivaceus*) is one of the economic important fish in China. Sexual dimorphism, especially the different growth rates and body sizes between two sexes, makes this fish a good model to investigate mechanisms responsible for such dimorphism for both fundamental questions in evolution and applied topics in aquaculture. However, the lack of “omics” data has hindered the process. The recent advent of RNA-sequencing technology provides a robust tool to further study characteristics of genomes of nonmodel species. Here, we performed *de novo* transcriptome sequencing for a double haploid Japanese flounder individual using Illumina sequencing. A single lane of paired-end sequencing produced more than 27 million reads. These reads were assembled into 107,318 nonredundant transcripts, half of which (51,563; 48.1%) were annotated by blastx to public protein database. A total of 1051 genes that had potential alternative splicings were detected by Chrysalis implemented in Trinity software. Four of 10 randomly picked genes were verified truly containing alternative splicing by cloning and Sanger sequencing. Notably, using a doubled haploid Japanese flounder individual allow us to analyze gene duplicates. In total, 3940 “single-nucleotide polymorphisms” were detected form 1859 genes, which may have happened gene duplicates. This study lays the foundation for structural and functional genomics studies in Japanese flounder.

Japanese flounder, *Paralichthys olivaceus*, belongs to genus Paralichthys, family Paralichthyidae, order Pleuronectiformes, and naturally distributes in the western Pacific (Yamada *et al.* 1995). Japanese flounder, loved by Asians, grows fast with good flavor, making it becomes one of main farmed fish in China, Japan, and Korea (Yamamoto 1999). As an aquaculture fish, studies on *P. olivaceus* focused on the development of genetic markers, construction of genetic linkage maps, and characterization of genes related to immunity and sex-determination (Sekino and Hara 2001; Coimbra *et al.* 2003). There is limited public data for this fish, *e.g.*, only 16,275 expressed sequence tag sequences are available. Until now, there has been no “omics” study on this fish.

Gynogenesis has been used for fish breeding, including *P. olivaceus* (Chourrout 1984; Komen *et al.* 1991; Tabata 1997). By suppression of the second polar body extrusion or the first mitotic cleavage, eggs fertilized with inactivated sperms achieve genome duplication and can develop into normal individuals (Arai 2001). The genetic background of gynogens is simple. The genome of meiotic diploids derives from the ooctye and the second polar body, and that of double haploids comes from duplication of oocyte, so gynogens, especially the double haploids, are good material for genetic research (Komen and Thorgaard 2007). Shikai *et al.* (Liu *et al.* 2012) identified 25,144 unique protein-encoding genes in catfish by RNA-Seq analysis of a doubled haploid homozygote, more than 14,000 of which were full-length transcript with complete open reading. Moreover, a total of 2659 unique genes were identified as putative duplicated genes because the corresponding transcripts harbored paralogous sequence variants (PSVs) or multisite variants (MSVs). In the present study, the individual fish we used for sequencing was double haploid.

During the past several years, high-throughput sequencing technology has provided great opportunities for “omics” research in an efficient and inexpensive way (Metzker 2009). This technology makes omics research possible for nonmodel species like *P. olivaceus*. Recently, the emerging RNA-sequencing (RNA-seq) goes further; it can access not only *de novo* transcriptome analysis but also quantitative analysis of the transcripts (Wang *et al.* 2009). The advent of RNA-seq sequencing facilities kinds of researches. For example, Nagalakshmi *et al.* (2008) applied RNA-seq to generating a high-resolution transcriptome map of the yeast genome. A highly integrated single-base resolution epigenome maps was generated in Arabidopsis by combining methylC-seq and RNA-seq (Lister *et al.* 2008). In another research, RNA-seq was used to identify the immune-relevant genes in marine fish (Xiang *et al.* 2010).

In the present study, we performed *de novo* transcriptome sequencing for a homozygous gynogenetic individual of *P. olivaceus* using Illumina RNA-seq technique. About 2.5 G of paired-end reads were produced and assembled into 107,318 nonredundant sequences, gene annotation was processed, and lots of function gene were identified. We also detected alternative splicing events in our data. This transcriptome data would be a valuable source and great facilitate for future research on this species.

## Materials and Methods

### Biological materials and RNA extraction

A 2-yr-old individual of double haploid *P. olivaceus* were kindly provided by Professor Feng You, Institute of Oceanology, Chinese Academy of Science. Samples of 19 tissues, including brain, gill, heart, liver, kidney, spleen, intestines (the end), muscle, and ovary, were collected. Tissues were flash-frozen in liquid nitrogen after dissecting and then stored at −80° until RNA extraction. Total RNA was extracted from these tissues using Trizol reagent (Invitrogen). The quantity and quality of total RNA was measured using a spectrophotometer (Bio-Spec-mini, Shimadzu, Japan) and gel electrophoresis. After DNA removal, equal amount of high-quality total RNA from each tissue was combined and sent out for commercial sequencing.

### Illumina sequencing

Sequencing was conducted commercially in Beijing Genomics Institute at Shenzhen (BGI, Shenzhen, China). A single lane was sequenced on HiSequation 2000 using a 90-bp, paired-end sequencing module.

### Sequence data analysis and assembly

The raw reads were first preprocessed by trimming adaptor sequences. Low-quality reads with Q value <13 also were removed using SolexaQA (version 1.13) (Cox *et al.* 2010). At last, high-quality reads with length more than 25 were retained. To obtain a comprehensive and reliable assembly, two assemblers, including SOAPdenovo (v1.03) and Trinity (version 2012-6-8), were used for *de novo* assembly. Afterward, the two sets of assemblies were combined to produce the final nonredundant assembly. As anticipated, some identical contigs were generated from two assembliers, and some identical transcripts were represented by multisimilar contigs, which both introduce redundancy. The cap3 (latest version) was used to remove redundancy and retain the longest possible contigs.

### Sequencing annotation

All the nonredundant transcripts merged from two assemblers were searched against the National Center for Biotechnology Information RefSeeq protein database and Uniprot/Swiss-Prot database using BLASTX with E-value < 1e-5. Then sequences with blast hits were carried out via Gene Ontology (GO) annotation with Blast2GO suite and KEGG analysis with the web tool KAAS (http://www.genome.jp/tools/kaas/).

### Identification of alternative splicing

Components with two or more sequences from Trinity assemblers were extracted by customized perl scripts. Primers were designed using Primer Premier 5. Polymerase chain reaction (PCR) amplifications were carried out in a volume of 25 μL containing approximately 10 ng of cNDA, 0.5 mM each dNTP, 0.2 μM each primer, 0.25 units of Taq DNA Polymerase, and the PCR buffer at 1 × concentration. An initial denaturation step of 3 min at 94° was followed by 35 cycles of of amplification (30 sec at 94°, 30 sec at 52° or 55°, which depends on the primers, and 30 sec at 72°) and a final elongation step of 10 min at 72°. The results of Sanger sequencing were removed vector sequences manually and aligned using Blastn and MegAlign in Lasergene packages.

### Identification transposable elements (TEs)

Putative TE were identified based on homology search. Our data sets were compared against RepBase 17.09 using tBLASTx with a threshold of 1e-5. The outputs were manually inspected, and significant matches to Simple Repeat, Pseudogene, and Integrated Virus were excluded.

### Detection of putative gene duplicates

Detection of putative gene duplicates was carried out according to Liu *et al.* (2012). Briefly, we mapped all the filtered reads (Q value ≥ 20) to the annotated unigenes with the similarity of 99%. The single-nucleotide polymorphism (“SNP”) must satisfy the following conditions: 1) each “SNP” position was supported by at least four reads, 2) the minimum number of variant alleles was two, and 3) minor allele frequency was at least 10%.

## Results and Discussion

### Sequencing and assembly

A mixed complementary DNA sample obtained from multiple tissue of a homozygous female Japanese flounder was prepared and sequenced using Illumina HiSeq 2000. A single lane of paired-end sequencing produced more than 27 million (M) raw reads, containing nearly 2.5 giga (G) nucleotides. After removing low-quality sequences (Q value <13), 24 M reads with a length of more than 25 bp were retained. The clean reads with the average length of 75.2 bp accounted for 88% of the raw reads.

Two different assemblers (SOAPdenovo and Trinity) were used to assemble clean reads to consensus (Li *et al.* 2010; Grabherr *et al.* 2011). By SOAPdenovo, three processes were performed to complete assembly. First, reads with overlap were assembled to contigs. And then paired-end reads were mapped to contigs to find out contigs derived from the same transcripts and the distance of them, the scaffolds were generated by filling gaps with N between these contigs. At last, paired-end reads were mapped to scaffolds to fill gaps. By these three steps, we obtained 119,370 scaffolds ranging from 150 bp to 9339 bp and with an average length of 469 bp and a N50 of 626 bp (Table 1). Among all the scaffold, 11,232 (9.41%) had a length of more than 1 kb. Regarding the length of the scaffolds with gaps, the proportion of N must be considered. The total length of scaffolds was 56 M bases with an N ratio of 0.09%.

**Table 1:** Summary of assemblies generated using two different assemblers for *P. olivaceus*

Assemblies	No. Contigs ≥150 bp	No. Contigs ≥1 kb	Avg. Contig Length, bp	N50, bp	Total size, M, bp
SOAPdenovo	119,370	11,232	469	626	56.0
Trinity	97,460	16,211	643	910	62.6
Final merged	107,318	19,167	646	1081	69.4

Similar to SOAPdenovo, Trinity also used de Bruijn graph algorithm and combined three independent software modules: Inchworm, Chrysalis, and Butterfly (Li *et al.* 2010; Grabherr *et al.* 2011). First, Inchworm assembled reads into the unique sequences of transcripts, and then Chyrysalis clustered the Inchworn contigs into clusters and construct complete de Bruijn graphs for each cluster. Finally, Butterfly then analyzed the paths taken by reads and paired of reads in the context of the corresponding de Bruijn graph and reported all plausible transcript sequences, resolving alternatively spliced isoforms and transcripts derived from paralogous genes. Trinity generated 97,460 contigs containing no N ranged from 201 bp to 10,284 bp (Table 1). The average length of these contigs was 643 bp with N50 of 910 bp. Of these 97,460 contigs, 16,211 (14.43%) had a length of more than 1 kb. The total length of contigs was 62.6 M bases.

As shown in Table 1, the SOAPdenovo assembly generated more sequences whereas Trinity assembly generated longer contigs and bigger total size. Considering the overall results, Trinity performed better than SOAPdenovo in this study. Previous studies compared different assemblers also showed that Trinity performed well across various conditions but took a longer running time (Zhao *et al.* 2011, 2013). Different assemblers may be suitable for different sequencing results. The safest way is to compare a few of different assemblers, and then select the one performed best. Here, we merged two sets of assemblies and got 107,318 nonredundant sequences with a longer average length and N50 (646 bp and 1081 bp, respectively) and a bigger total size (69.4 Mbp). The subsequent annotation were all based on the merged sequence set.

### Sequencing annotation

**Figure 1:**
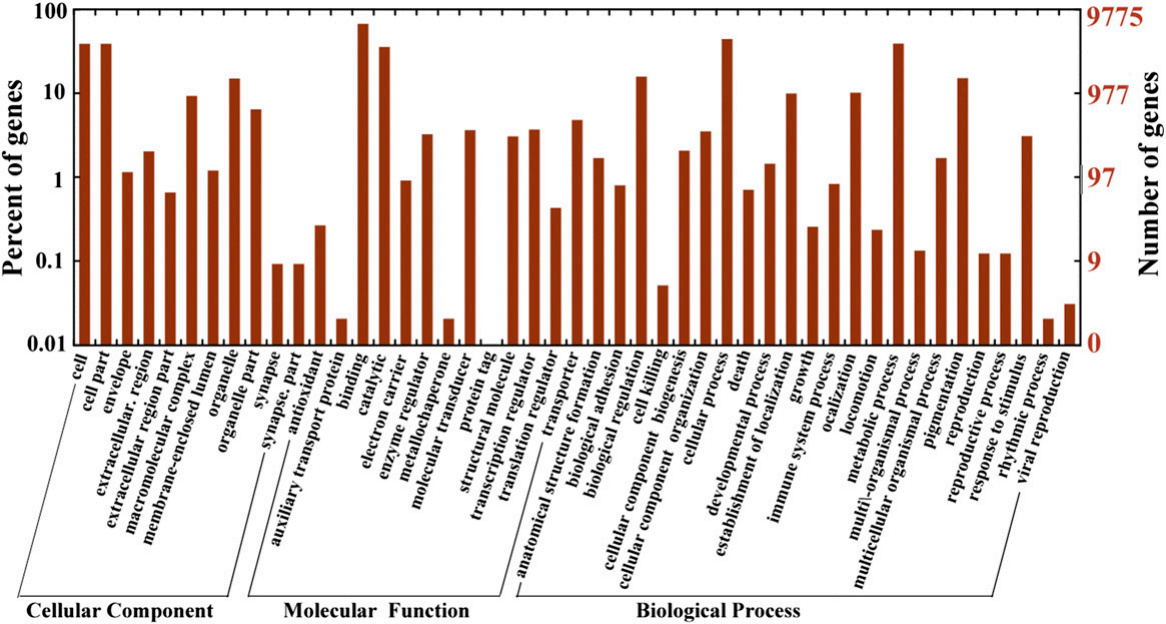
Gene ontology (GO) representations for *P. olivaceus*. The most representative two GO terms are cell and cell part in Cellular Component, binding and catalytic in Molecular Function, and cellular process and metabolic process in Biological Process.

We used several complementary approaches to annotate the assembled sequences. First, unigenes were compared against the public protein databases (nr) using BLASTX (E-value < 1e-5) (Camacho *et al.* 2009). By similarity search, nearly half of sequences (51,563, 48.1%) returned protein coding information (Supporting Information, File S1). Second, unigenes that had matches with nr database were assigned for biological process (GO: 0008150), cellular component (GO: 0005575), and molecular function (GO: 0003674) by GO annotation (Figure 1 and File S2) (Ashburner *et al.* 2000). In total, 17,833 (34.98%) sequences got 37,541 different GO terms. Third, the KEGG (Kyoto Encyclopedia of Genes and Genomes) pathway approach for higher order functional annotation was implemented using the web tool KASS. A total of 7811 sequences got KO numbers and were mapped to 310 different pathways. Interestingly, Pathways in cancer (05200) was the best represented pathway to which 163 unigenes were mapped (Figure S1).

### Identification of alternative splicing

Alternative splicing allows organisms with relatively less genes to encode more proteins. It is time- and labor-consuming to discover alternative splicing by cloning genes and resequencing. RNA-Seq is a fast and efficient way to identify alternative splicing. Chrysalis, the second step of Trinity, clusters minimally overlapping Inchworm contigs into sets of connected components that comprise alternative splice forms or closely related paralogs (Grabherr *et al.* 2011). We first annotated the data set resulting from Trinity with BlastX, and then searched the components with two or more sequences in the annotated unigene. In total, 1051 we found components in which potential alternative splicing should exist (File S3).

**Figure 2:**
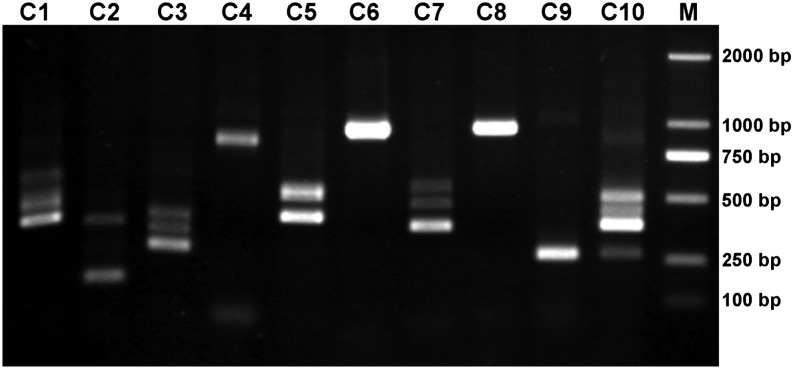
Amplification result of 10primer pairs. M D2000.

To verify these 1051 components truly containing alternative splicing, we randomly selected 10 components and designed primer pairs (named C1-C10; Table 2) that stepped over the difference of sequences from the same components. All 10 primer pairs successfully amplified products, and six of them amplified more than one products (Figure 2). A total of 26 samples were collected and sent to Sanger sequencing, in which 24 were successfully sequenced. It is worthy to note that primer C6 also amplified two products and they happened to have the same length, so there was only one band on gel. By sequence alignment, we found that the components according to primers C1, C5, C6, and C10 were truly alternative splice isoforms. Though primer pairs C2, C3, and C7 got multiple products, only one was target product and the others were all non-specific amplification.

**Table 2:** Information of 10 primer pairs used for verifying alternative splicing

Pirmer ID	Primer Sequences	Tm, °C	Sequence ID	Sequence Annotation
AC1	Fw-AATCCAGCGTTCTTTACCA	53.1	comp69204_c0	ATP synthase coupling factor 6
	Rv-AGCAAGGAAGCCGCCATCT	62.1		
AC2	Fw-GGCTGTCATTATCCTGCCTCA	60.0	comp72080_c0	Peptide chain release factor 1
	Rv-CGGTGATGCCTGTTGGGAG	61.7		
AC3	Fw-CAACAGGTCTGAGGGAGGC	57.3	comp72304_c0	Sequestosome-1-like
	Rv-TTCTGGTTATGGCATTGGT	53.4		
AC4	Fw-CCACTACAAATGGCACTTCG	56.5	comp73211_c0	Ankyrin-1
	Rv-AAACCTCATCACCGTAGCG	55.8		
AC5	Fw-AAAGGAGACGGGCTACATC	54.0	comp73262_c0	Protein polybromo-1-like
	Rv-CTGCTGGGTTGTCTTGTGC	56.8		
AC6	Fw-GTGGCGTTCAGATGTTAGAC	52.6	comp73364_c0	Multiple C2 and transmembrane domain-containing protein 2-like
	Rv-CAGGATGACAATGGCAGAG	53.7		
AC7	Rv-Fw-TCTACTCGGTTGGCTTCGT	55.5	comp73373_c0	Protein tyrosine phosphatase-like A domain containing 2-like
	Rv-GTTTAGTGTCGTCGGCTCA	53.9		
AC8	Fw-ACCGCAATGTGGTCGTTAG	56.5	comp73575_c0	Hypothetical protein LOC100703628
	Rv-CTCCCATACACTGAGATATTACTTG	55.3		
AC9	Fw-CCCACTACACCCTGACCAC	55.0	comp73625_c0	Cytochrome P450 2J2-like
	Rv-CATTCCTCCTGGTGCTTCT	54.5		
AC10	Fw-GCGTTACATTCCACCTCACC	57.6	comp73862_c0	Putative ATP-dependent RNA helicase an3-like isoform 2
	Rv-AGGAGCAGTGGGCTTTGAC	57.3		

### Identification of TEs

**Figure 3:**
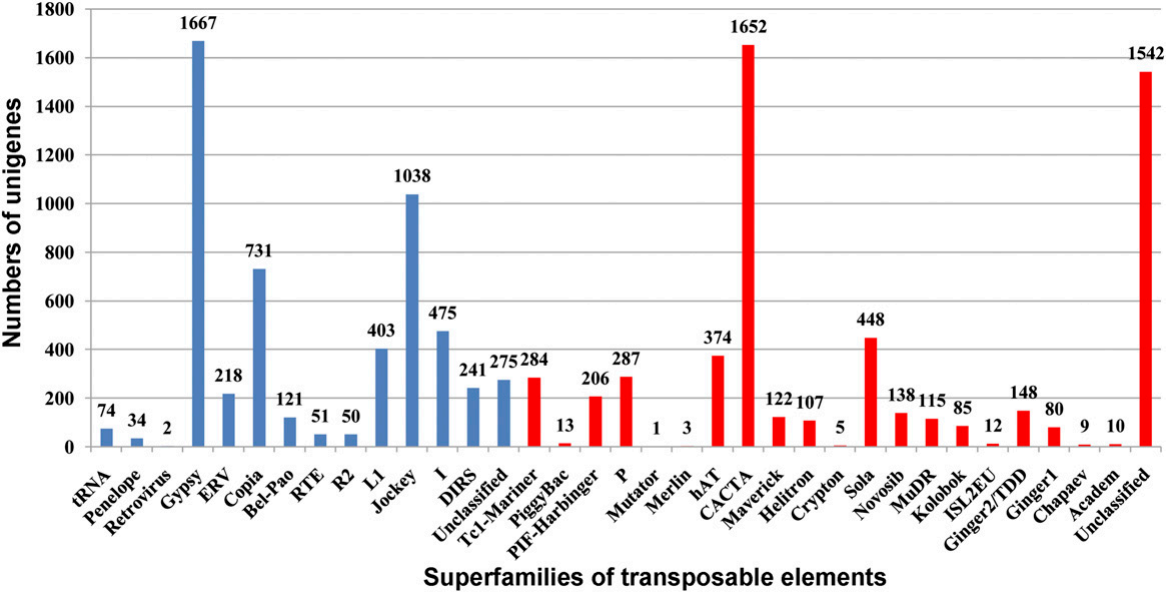
Abundance distribution of transposable elements in the unigenes of *P. olivaceus*. The blue bars represent retroelements, whereas the red bars represent DNA transposons.

TEs can be divided into two general classes. Class I, termed retroelements, all transpose via an RNA intermediate. Class II, termed DNA transposons, are those that can directly manipulate DNA to propagate themselves into another place within a genome. A search of our transcriptome data revealed that 11,021 sequences contained putative TEs, of which 5380 TEs belonged to retroelements and 5641 TEs belonged to DNA transposons (Figure 3 and File S4). The most frequent retroelements was Gypsy (1667, 30.1%), followed by Jockey (1038, 19.3%), and Copia (731, 13.6%), whereas the most frequent DNA transposons was CACTA (1652, 29.3%), followed by Sola (448, 7.9%), and hAT (374, 6.6%).

### Identification of gene duplicates

**Figure 4:**
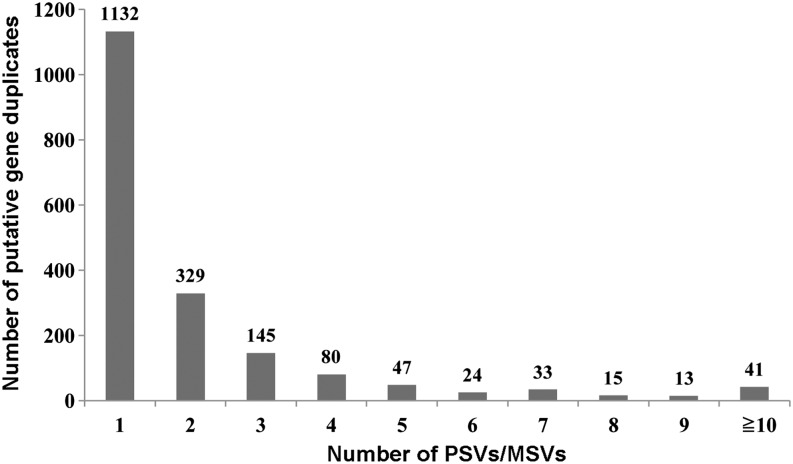
Detection of putative *P. olivaceus* gene duplicates. X-axis represents the number of PSVs or MSVs detected, whereas the Y-axis is the number of putative duplicated genes in catfish that contained the PSVs or MSVs. MSV, multisite variant; PSV, paralogous sequence variant.

Teleost fish, including Pleuronectiformes, have experienced genome duplication three times (Taylor *et al.* 2003; Volff 2004). This process increased the complexity of the fish genome and the difficulty of sequence assembly and annotation. Duplicated regions contain PSVs and MSVs, which hindered SNP identification (Fredman *et al.* 2004). Conversely, this also provided a method of identifying gene duplicate by identifying “SNP.” In this work, we used a doubled haploid Japanese flounder individual, which made this method possible as it had two sets of identical chromosomes. Therefore, we detected SNPs in the 51,563 annotated unigenes and a total of 3,940 “SNPs” were detected from 1859 unigenes (File S5). Most of transcripts (60.9%) were detected having only one “SNPs,” whereas a few of transcripts (6.8%) containing more than five “SNPs” (Figure 4). These “SNPs” might represent PSVs or MSVs as well as these transcripts might exist potential duplicated gene.

In this study, we performed *de novo* transcriptome sequencing for an economically important fish, Japanese flounder, *Paralichthys olivaceus*. Illumina sequencing produced more than 27 million reads. These reads were assembled into 107,318 nonredundant transcripts, and nearly half of them (51,563, 48.1%) were annotated. Alternative splices were detected and extra experiment were carried out to evaluate it. Notably, a doubled haploid Japanese flounder individual allowed us to analyze gene duplicates. In total, 1859 genes may have happened gene duplicates. This study will contribute significantly toward structural and functional genomics studies in Japanese flounder.

## Supplementary Material

Supporting Information
