# Supplementary material for: Detection of Alternative Splice and Gene Duplication by RNA Sequencing in Japanese Flounder, Paralichthys olivaceus
Source: G3 (Bethesda). 2014 Nov 5;4(12):2419–24. doi: 10.1534/g3.114.012138 (PMC4267937; doi:10.1534/g3.114.012138)
Supplement: Supporting Information [file supp_4_12_2419__index.html]

Detection of Alternative Splice and Gene Duplication by RNA Sequencing in Japanese Flounder, Paralichthys olivaceus — Supporting Information 

# Detection of Alternative Splice and Gene Duplication by RNA Sequencing in Japanese Flounder, *Paralichthys olivaceus*

## Supporting Information for Wang *et al.*, 2014

**Files in this Data Supplement:**

- Figure S1 - Pathways in cancer annotation information in Japanese flounder transcriptome. (PDF, 448 KB)
- File S1 - BLASTX annotation for Japanese flounder transcriptome. (.xlsx, 4 MB)
- File S2 - GO annotation for Japanese flounder transcriptome. (.xlsx, 4 MB)
- File S3 - List of transcripts may have alternative splice isoform. (.txt, 35 KB)
- File S4 - Transposable elements in Japanese flounder. (.xlsx, 909 KB)
- File S5 - Information of transcripts may represent duplicated gene. (.xlsx, 217 KB)
