# Supplementary material for: Detection of Alternative Splice and Gene Duplication by RNA Sequencing in Japanese Flounder, Paralichthys olivaceus
Source: G3 (Bethesda). 2014 Nov 5;4(12):2419–24. doi: 10.1534/g3.114.012138 (PMC4267937; doi:10.1534/g3.114.012138)
Supplement: Supporting Information [file supp_g3.114.012138_FigureS1.pdf]

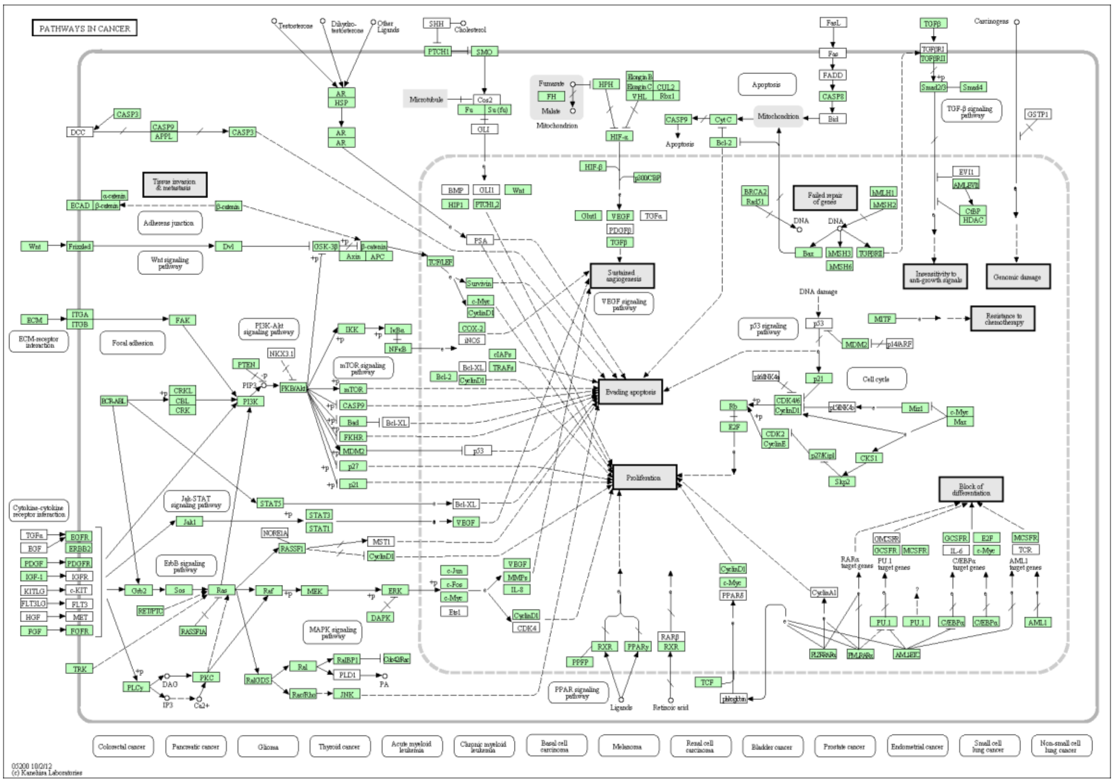

Figure S1 Pathways in cancer annotation information in Japanese flounder transcriptome. The green blocks mean genes identified in the Japanese flounder transcriptome.
